# Supplementary material for: Seroprevalence of Measles Antibodies in a Highly MMR-Vaccinated Population
Source: Vaccines (Basel). 2022 Nov 3;10(11):1859. doi: 10.3390/vaccines10111859 (PMC9698789; doi:10.3390/vaccines10111859)
Supplement: Supplementary file 1 [file vaccines-10-01859-s001.zip › vaccines-1982984-supplementary.pdf]

Supplementary Materials

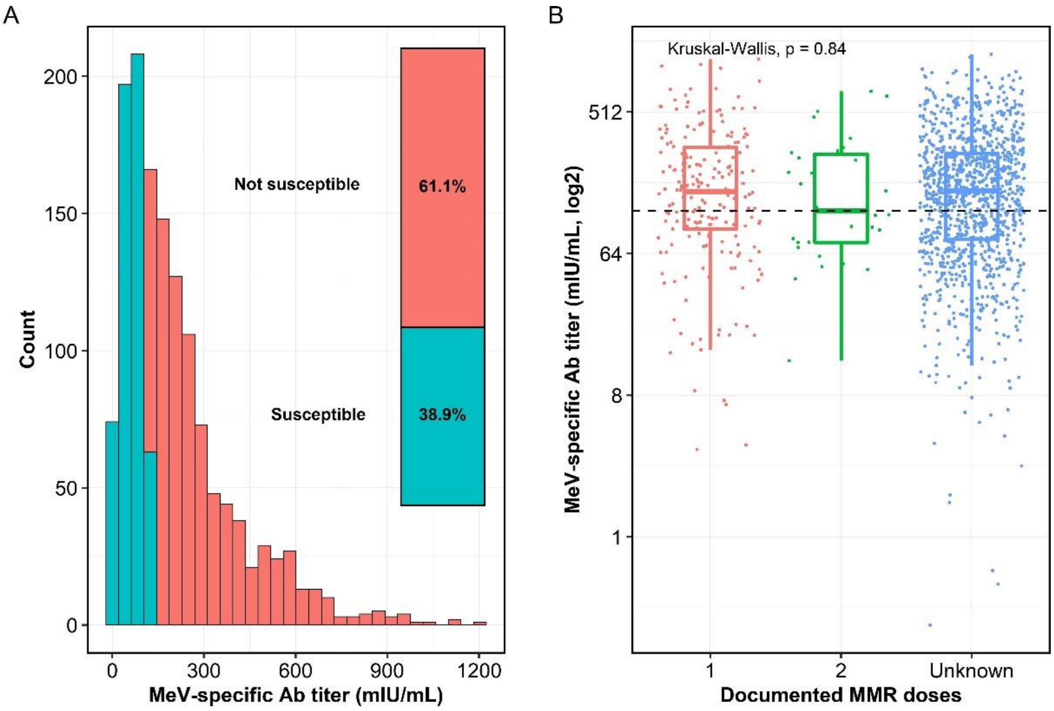

Supplementary Figure S1. Measles-specific IgG antibodies. (A) Distribution of measles-specific IgG antibody titer. Measles-specific IgG titer was calculated using the Third WHO International Standard Serum for Anti-measles serum (NIBSC code: 97/648) and expressed in mIU/mL. On the basis of the IgG titer, 851 (61.1%) and 542 (38.9%) serum samples were classified as not susceptible and susceptible to measles, respectively, using a pre-defined protective titer of 120 mIU/mL as a threshold (Figure S1A inset). There was no significant difference in the IgG titer among subjects with one-dose, two-dose and unknown-dose MMR vaccination. Dashed line in Figure S1B represents the protective threshold titer of 120 mIU/mL.
